# AIR-LEISH: A Dataset of Giemsa-Stained Microscopy Images for AI-based *Leishmania* amastigotes Detection

**DOI:** 10.1038/s41597-026-06676-8

**Published:** 2026-02-02

**Authors:** Rafeh Oualha, Nesrine Fekih-Romdhane, Donia Driss, Yosser Zina Abdelkrim, Ikram Guizani, Emna Harigua-Souiai

**Affiliations:** 1 Laboratory of Molecular Epidemiology and Experimental Pathology - LR16IPT04, Institut Pasteur de Tunis, Université de Tunis El Manar, Tunis, Tunisia; 2 Mediterranean Institute of Technology (MedTech), South Mediterranean University (SMU), Tunis, Tunisia

**Keywords:** Data processing, Cellular imaging

## Abstract

Leishmaniases is a parasitic disease caused by the *Leishmania* parasite, transmitted by sandflies, affecting millions worldwide. Microscopic examination remains the standard method for detecting and quantifying intracellular parasite burden in leishmaniases research and Drug Discovery. This process is time-consuming and requires specific expertise. While Artificial Intelligence shows promise in automating this task, progress is limited by the lack of annotated datasets. To address this gap, we present AIR-LEISH, a dataset of 180 Giemsa-stained microscopic images with expert annotations containing 8,140 *Leishmania* amastigotes and 1511 macrophages. Images corresponded to samples from two infection models. The dataset was annotated to facilitate AI-based object detection and image segmentation tasks. We further demonstrated the potential of this dataset through training and testing two state-of-the-art architectures, namely YOLOv8 and U-Net. Both models demonstrated promising performance for automatic classification, detection and counting of amastigotes. The dataset is freely available on the Zenodo platform to accelerate the development of AI-based tools, facilitate advances in leishmaniases research and support collaborative initiatives for public health.

## Background & Summary

Leishmaniases are neglected parasitic diseases caused by protozoa of the genus *Leishmania*, transmitted to humans through the bite of infected female phlebotomine sandflies. They remain a major public health concern in many endemic regions, affecting more than 12 million people globally and causing more than 30,000 deaths annually^1^. Several clinical forms exist ranging from cutaneous lesions (CL) to visceral leishmaniasis (VL). Currently, there is no vaccine available for these diseases, and treatment options are often limited, associated with significant toxicity, high cost, and increasing resistance to current therapies. These challenges highlight the urgent need for rapid diagnostic methods, novel therapeutic strategies and a deeper understanding of host-pathogen interactions.

In the context of leishmaniases, diagnosis still relies on clinical manifestations combined with parasitological or serological tests^1^. While molecular detection methods such as PCR provide higher sensitivity and are being adopted in specialized laboratories^2^, microscopic examination remains essential as a practical diagnostic and parasite quantification standard in most research laboratories in the global south and in low resources settings^3–5^. However, in the frame of research projects, *e.g*. drug discovery, that involve several conditions examination and parasite load assessment through manual counting, this technique appears to be time consuming, labor intensive, and operator-dependent, which can affect the reproducibility of the results. In recent years, artificial intelligence (AI) and machine learning (ML) have demonstrated strong potential for automating such analyses^3^, particularly through object detection^4,6^ and image segmentation approaches^5,7,8^. Despite these advancements, AI-based tools remain limited by the reduced availability of pathogen-specific AI-ready datasets^9,10^. Publicly accessible image datasets have recently emerged for some parasitic diseases, including Trypanosomiasis^11^ and Malaria^12,13^ among others. In the case of leishmaniases, few open-access datasets exist. These mainly cover the promastigote form of the parasite^14^ or *in vitro* infected macrophages^10^, or correspond to clinical samples from patients^5,15^, which are only available upon request^4,5,10,15^. Although previous studies claimed that datasets were available, in-depth examination of the manuscripts examination, the provided links were found to be inactive and the datasets were no longer accessible, revealing sustainability issues^4,6,8,16^. To the best of our knowledge, no publicly available dataset currently exists covering the intracellular amastigote form, which is the clinically relevant stage in human hosts. Such datasets are necessary to train deep learning models in the context of parasite detection and identification.

To bridge this gap, we herein introduced the AIR-LEISH dataset^17^, a curated collection of Giemsa-stained microscopy images of intracellular *Leishmania* amastigotes within experimentally infected macrophages. This dataset was generated using two complementary *in vitro* infection models. The first model, which involved THP-1-derived macrophages infected with *L. major* parasites, offers a standardized and scalable system for antileishmanial drug screening. The second model, which used primary human monocyte-derived macrophages infected with *L. infantum* parasites, provides a more physiologically relevant context to investigate host-pathogen interactions. Through these models, we covered different morphological aspects of *Leishmania* parasites through two different species, as well as cell aspects through two cell types. In both cases, infections were conducted under controlled experimental conditions and imaged using standard light microscopes. All images were manually annotated by experts to indicate the location of host cells, nuclei and intracellular amastigotes. The dataset was then structured into training, validation, and test sets. Annotations were provided in a format that enables the training of deep learning models for object detection, and for segmentation. A validation step consisted in training the YOLO8 and U-Net models to detect and count amastigotes within the images.

The AIR-LEISH^17^ dataset was designed to support a wide range of applications, from Drug Discovery and pathogenesis research to the development of diagnostic-supporting tools, at the longer term. By making this dataset publicly and freely available on the Zenodo platform, we aim to foster the development of computational tools that can accelerate image-based research on leishmaniases. This resource is highly valuable for studies combining experimental parasitology and AI approaches. These shall provide supporting tools to minimize manual effort, thus enabling fast and accurate analysis of microscopy images, especially in low resource settings. Furthermore, it benefits research teams that may not have access to large datasets or the necessary expertise for sample preparation and annotation.

## Methods

### Sample preparation

The dataset was generated from microscopy images of experimentally infected macrophages with *Leishmania* parasites, using two distinct infection models established in our laboratory. The first model involved THP-1 macrophages infected with *L. major* and was established as part of our drug discovery pipeline, specifically for the identification and validation of novel therapeutic molecules^14–17^. The second model, established to investigate host-pathogen interactions and mechanisms of action, utilized pre-existing microscope slides from previous laboratory experiments. These slides corresponded to human monocyte-derived macrophages (MDMs) from healthy adult volunteers, experimentally infected with *L. infantum*. Peripheral Blood Mononuclear Cells (PBMCs) were isolated from the donors, who participated voluntarily in the study after providing written informed consent for sample collection and use in research. No personal or identifiable data were collected or shared. The original study involving these MDMs was ethically approved by the Ethics Committee of the Institut Pasteur de Tunis (reference number: 2018/07/I/LR11IPT04).

In the first model, THP-1 cells, a human monocyte-derived cell line, were cultured in RPMI 1640/GlutaMAX medium (Gibco) supplemented with penicillin (100 U/mL), streptomycin (100 μg/mL), and 10% fetal bovine serum (FBS; Gibco), and maintained at 37 °C in a 5% CO₂. THP-1 cells (4 × 10⁴ per well) were seeded in Lab-Tek™ II chamber slides and differentiated into adherent macrophage-like cells by treatment with 25 ng/mL of PMA (phorbol 12-myristate 13-acetate) (Sigma-Aldrich) for 24 h. Adherent macrophages were infected with stationary phase promastigotes of *L. major* strain (EMPA-12) at a parasite-to-cell ratio 10:1 (10 parasites per macrophage). After 24 h of infection, non-internalized extracellular parasites were removed by washing and macrophages were stained using the RAL 555 kit (RAL Diagnostics) as previously described^18–21^.

In the second model, peripheral blood human mononuclear cells (PBMCs) were isolated from healthy donor buffy coats by Ficoll-Paque density centrifugation, as previously described^22^. Cells (10⁶ cells/mL) were then washed and resuspended in RPMI 1640/GlutaMAX medium (Gibco) containing penicillin (100 U/mL), streptomycin (100 µg/mL) and 10% heat-inactivated human AB serum, then seeded into gelatin-coated T75 flasks. After 2 hours of incubation at 37 °C in a humidified atmosphere containing 5% CO₂, non-adherent cells were removed. Adherent monocytes were detached using 0.05% Trypsin-EDTA (1 × ) (Gibco), following the manufacturer’s recommendations. Collected monocytes were counted and seeded at 5 × 10^5^ cells per well into a Lab-Tek™ II chamber slide. After 6 days of incubation under the same conditions, monocytes differentiated into macrophages (MDMs) and were infected with stationary phase promastigotes of *L. infantum* strain (LV50) at a parasite-to-cell ratio 15:1 (10 parasites per macrophage). After 24 h of infection, extracellular parasites were removed and cells were fixed and stained using May-Grünwald solution (RAL Diagnostics).

### Microscopy data acquisition

**Fig. 1:**
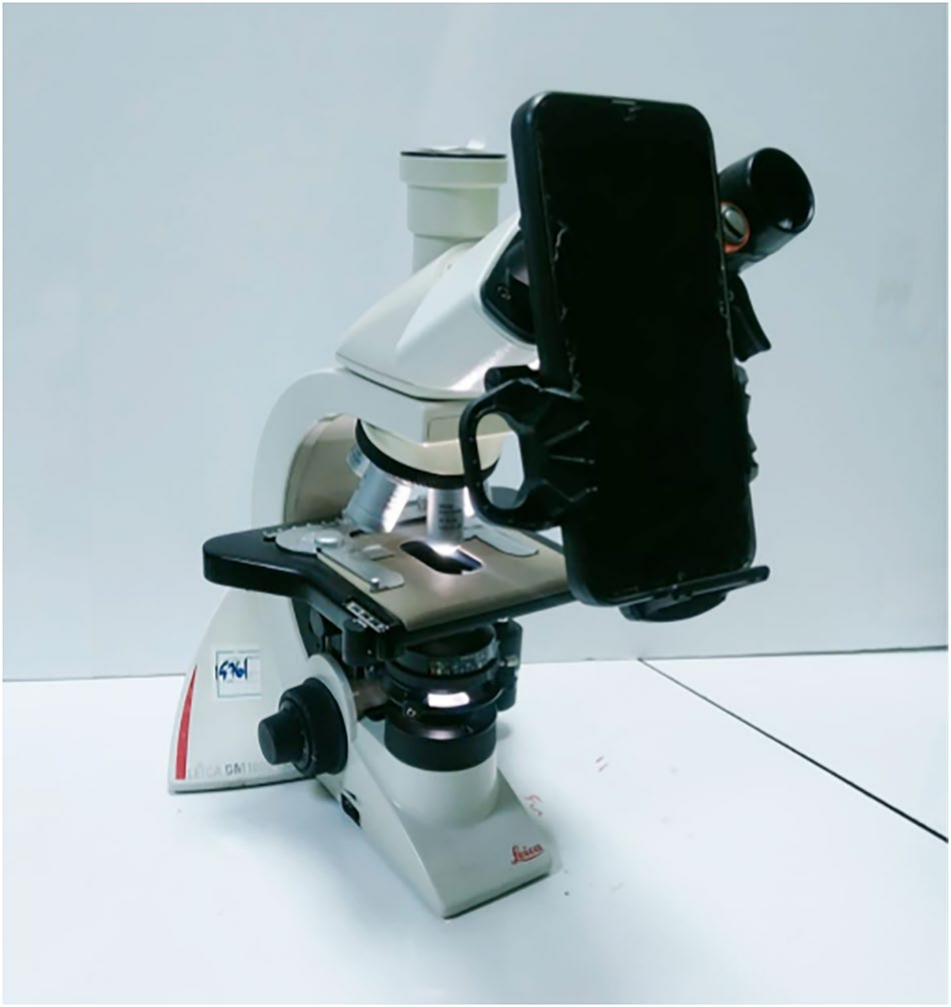
The microscopy setup used for image acquisition. A light microscope (LEICA DM1000 LED) was used in combination with a smartphone mounted on a NexYZ 3-Axis Universal Smartphone Adapter.

We used a LEICA DM1000 LED light microscope under a 100 × objective that was not equipped with an integrated camera. Images were acquired, under oil-immersion, using the front camera of a mobile phone, Samsung Galaxy A35 5 G (model SM-A356E/DSN), with a 50-megapixel (MP) resolution. The smartphone was mounted on a NexYZ 3-Axis Universal Smartphone Adapter (Celestron) to ensure stability and uniformity of the focus during image acquisition. The complete apparatus is illustrated in Fig. 1. In total, 180 images were captured with an average of six images per Lab-Tek™ II chamber slide. These corresponded to 90 images of infected THP-1 derived macrophages herein denoted as Set1, and 90 images of infected Monocytes Derived Macrophages (MDMs) herein denoted as Set2 (see Sample preparation section). No filters or image processing were applied during image acquisition. All images were resized to a standard size of 1,844 × 2,709 pixels, stored in PNG format. All images were further transferred from the mobile phone to the computation station using Google Drive to preserve quality during storage and sharing, and to avoid lossy compression artifacts.

### Image annotation

#### Manual annotation using Roboflow

**Fig. 2:**
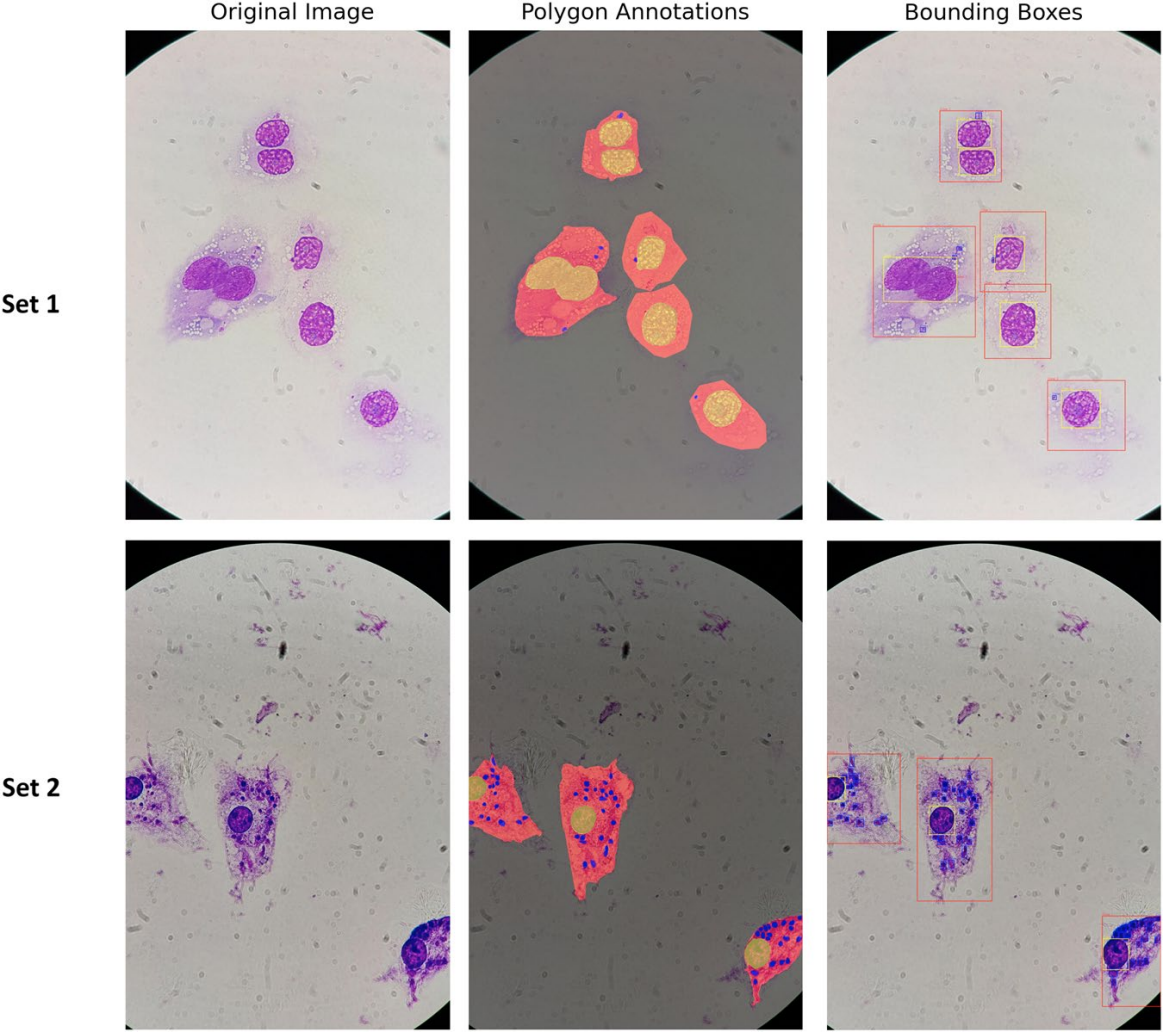
Examples of annotated images from both sets. The top row shows an image from Set1 (THP-1 infected with *L. major*). The bottom row shows an image from Set2 (MDMs infected with *L. infantum*). For each set, presented from left to right: the original image, the polygon annotations useful for segmentation tasks, and the bounding boxes useful for object detection.

Following image acquisition, manual annotation of the microscopy images was performed using the open-source version of the Roboflow platform (https://roboflow.com), which offers a detailed object labeling tool. Annotations were carried out by outlining relevant structures using polygonal shapes directly on the images, as illustrated in Fig. 2. Four object classes were considered: amastigotes, nuclei, host cells and background. The kinetoplast, a defining feature of *Leishmania* parasites, was not annotated separately because its visibility varied across Giemsa-stained images due to optical limits. Therefore, amastigotes were annotated as a unique instance/object. After completion, annotations were exported in Microsoft Common Objects in Context (MS COCO) format and corresponding text files, compatible with both segmentation and object detection tasks.

### Expert validation

To ensure high-quality ground truth annotations, we proceeded in two steps. First, a domain expert carefully annotated all biological entities within the images into: host cell, nucleus, or amastigote. In the second step, an AI engineer refined this annotation pixel-wise. Through this approach, a detailed delimitation of all biological entities was obtained. All relevant structures, including small or overlapping amastigotes, were included to ensure annotation completeness and accuracy. To evaluate the consistency of the annotation process, an inter-annotator agreement analysis was performed. The overlap between the two sets of annotations was quantified using the Dice Similarity Coefficient (DSC), defined as Eq. (1):

1 $$DSC=\frac{1}{4}{\sum }_{c=1}^{4}\frac{2{\sum }_{i=1}^{N}\,{p}_{ic}{g}_{ic}}{{\sum }_{i=1}^{N}\,{p}_{ic}+{\sum }_{i=1}^{N}\,{g}_{ic}}$$

where:

- ${p}_{{ic}}:$ prediction for class $c$ at pixel $i$
- ${g}_{{ic}}:$ ground truth for class $c$ at pixel $i$
- $N$: total number of pixel

The average Dice score across all annotated images was 95.6%, confirming the reliability of the manual annotation procedure. The final dataset contained 180 annotated images.

### Mask generation

We generated masks tailored for semantic segmentation, a technique that assigns a class label to each pixel in the image. Unlike instance segmentation, where individual objects are distinguished, semantic segmentation assigns a class label to each pixel without differentiating between object instances of the same class. A unique numerical label was assigned to each class: amastigotes, host cells, and nuclei. The resulting masks were then stored in PNG format to preserve each pixel value and avoid data loss (Fig. 2).

## Data Record

The dataset has been uploaded to the Zenodo platform^17^. It is distributed under the CC-BY 4.0 Licence, and was made openly accessible to foster scientific discovery and the development of computational tools. It can be downloaded as a compressed folder (.zip) through the Zenodo platform under the 10.5281/zenodo.17384855.

**Fig. 3:**
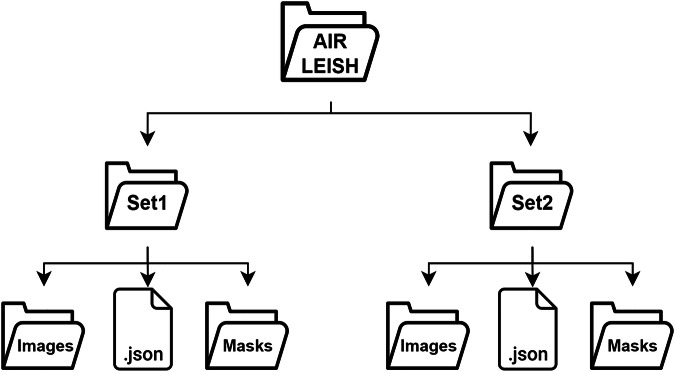
Directory structure for the AIR-LEISH dataset under the Zenodo platform. The dataset was organized into two primary directories: Set1 and Set2 which corresponded to two distinct infection models. Each subdirectory included a folder containing the original microscopy images, a Masks folder with pixel-level segmentation masks, and a JSON file formatted according to the MS COCO standard.

The dataset was organized into two primary directories: Set1 and Set2 which corresponded to the two distinct infection models described in Section 1, with 90 annotated microscopy images in each set.. Within each subdirectory, we included a folder containing the original microscopy images, a Masks folder with pixel-level segmentation masks, and a JSON file formatted according to the MS COCO standard. The latter format was used to ensure compatibility with models such as U-Net and Mask R-CNN. File naming followed a standardized convention: <20250328_CCimage_id>.png for the first set (Set1) and <20250203_CFimage_id>.png for the second set (Set2), facilitating seamless pairing of images with their corresponding annotations. Figure 3 illustrates the overall dataset directory tree. This separation of datasets, combined with a unified folder structure, was implemented to enable comparative evaluation under varying experimental and staining conditions.

## Technical Validation

### Data statistics

**Fig. 4:**
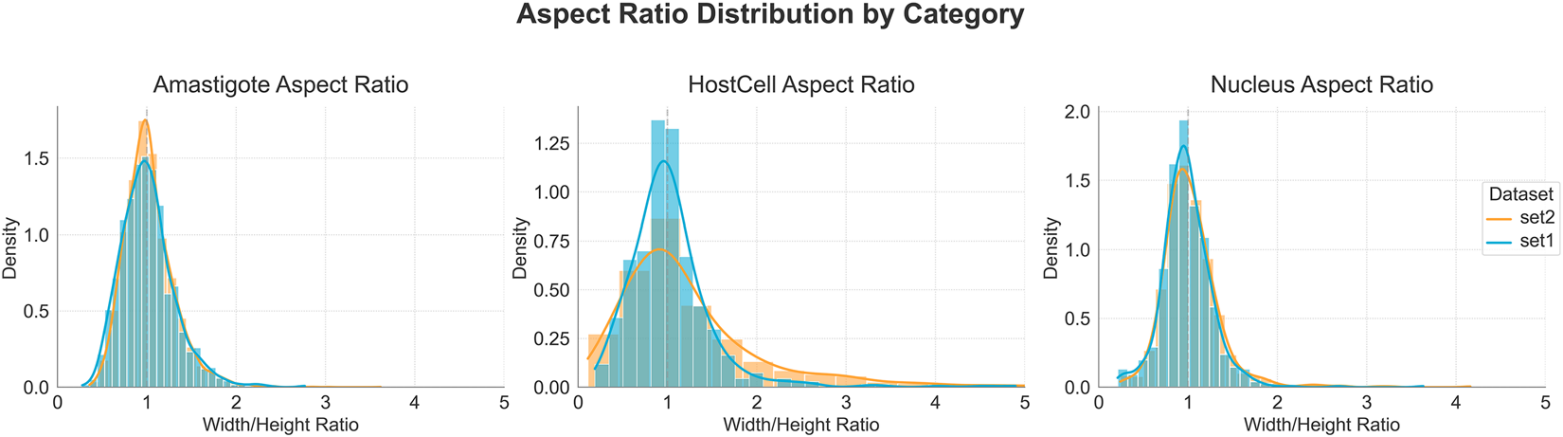
Aspect ratio distribution by category in Set1 and Set2. Density plots showing the width-to-height ratio distributions for amastigotes, host cells, and nuclei, demonstrating consistency between the two datasets despite biological and imaging variability.

To evaluate the quality of our data, we performed a statistical analysis of both subsets, Set1 and Set2, separately. We focused on four main aspects: the aspect Ratio distribution by category, host cell infection status, the category distributions and the area of the objects. First, we assessed the aspect ratio distributions, calculated as the width-to-height ratio for the three annotated classes: amastigotes, host cells, and nuclei. The distributions were consistent across the two datasets, despite biological and imaging variability (Fig. 4). Amastigotes, in particular, showed remarkable consistency with an average aspect ratio of 1.01 ± 0.15 and 1.03 ± 0.12 for Set1 and Set2, respectively. Additionally, 89% of the ratio values varied between 0.8 and 1.2, consistent with their circular/oval morphology. Conversely, host cells exhibited significant variability, reflecting biological diversity between THP-1-derived macrophages (Set2) and human monocyte-derived macrophages (MDMs) (Set1). Nuclei also maintained tight distributions (IQR: 0.85–1.15) with outliers (<0.5 or >2) resulting from partial object annotations at the boundaries of the images.

**Fig. 5:**
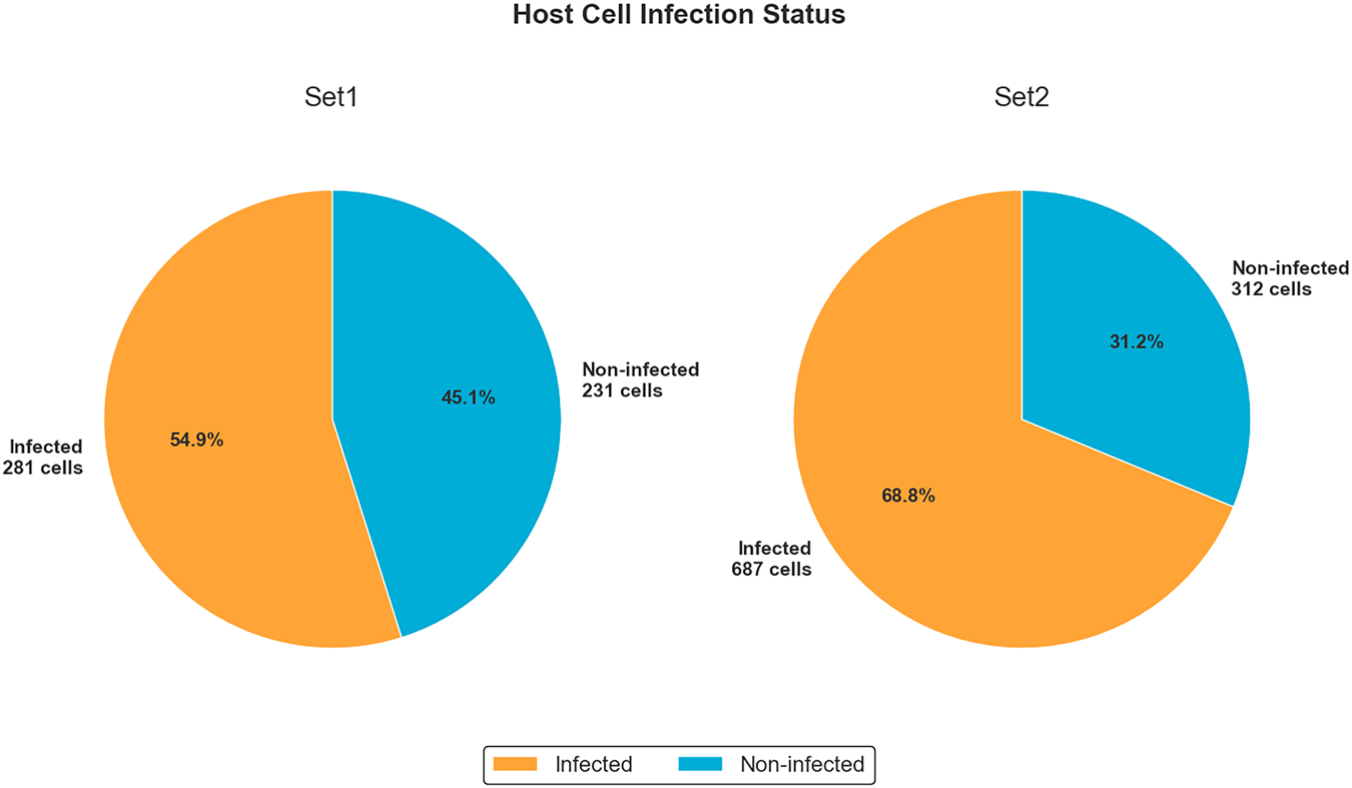
Percentage of infected and non-infected host cells in Set1 and Set2. Pie charts comparing infection status across datasets, showing Set2 had significantly higher infection rates compared to Set1. Proportional differences highlight potential biological variations between sample sets.

Set1 and Set2 presented different infection status as per the experimental design. In Set1, 54.9% of host cells were infected, while in Set2, the infection rate was higher, reaching 68.8% (Fig. 5). This 1.25-fold likely reflected biological differences between the *in vitro* infection models used herein, namely, differences in host cell type and number, parasite strain, cell to parasite ratios, parasite load, as well as potential annotation bias due to cell clustering. Subsequently, Set1 and Set2 presented different object densities (Fig. 5).

**Fig. 6:**
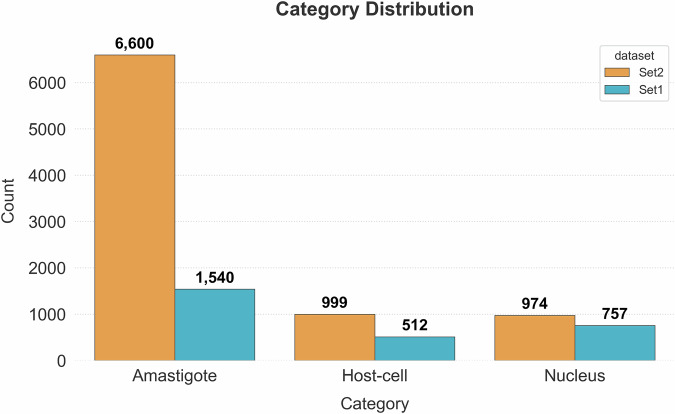
Category distribution of annotated objects in the AIR-LEISH dataset. The bar chart illustrates the total number of each annotated category: amastigotes, host cells, and nucleus, across the two sets (Set1 and Set2).

As a final figure, Set2 contained 6,600 amastigotes thus presenting 4.2-fold higher than Set1, which only contained 1,540 amastigotes. Interestingly, Set1 presented a lower count of host cells compared to nuclei, consistent with our earlier observation that adjacent host cells were fused during annotation, while nuclei could be distinctly labeled. Additionally, some host cells appeared binucleated, which further contributed to the discrepancy between the number of host cells and nuclei in Set1 (Fig. 2). Altogether, the dataset included a total of 8,140 annotated amastigotes, along with 1511 host cells and 1731 nuclei (Fig. 6). This collection offered a rich resource for training and evaluating AI models on cell and amastigotes detection.

**Fig. 7:**
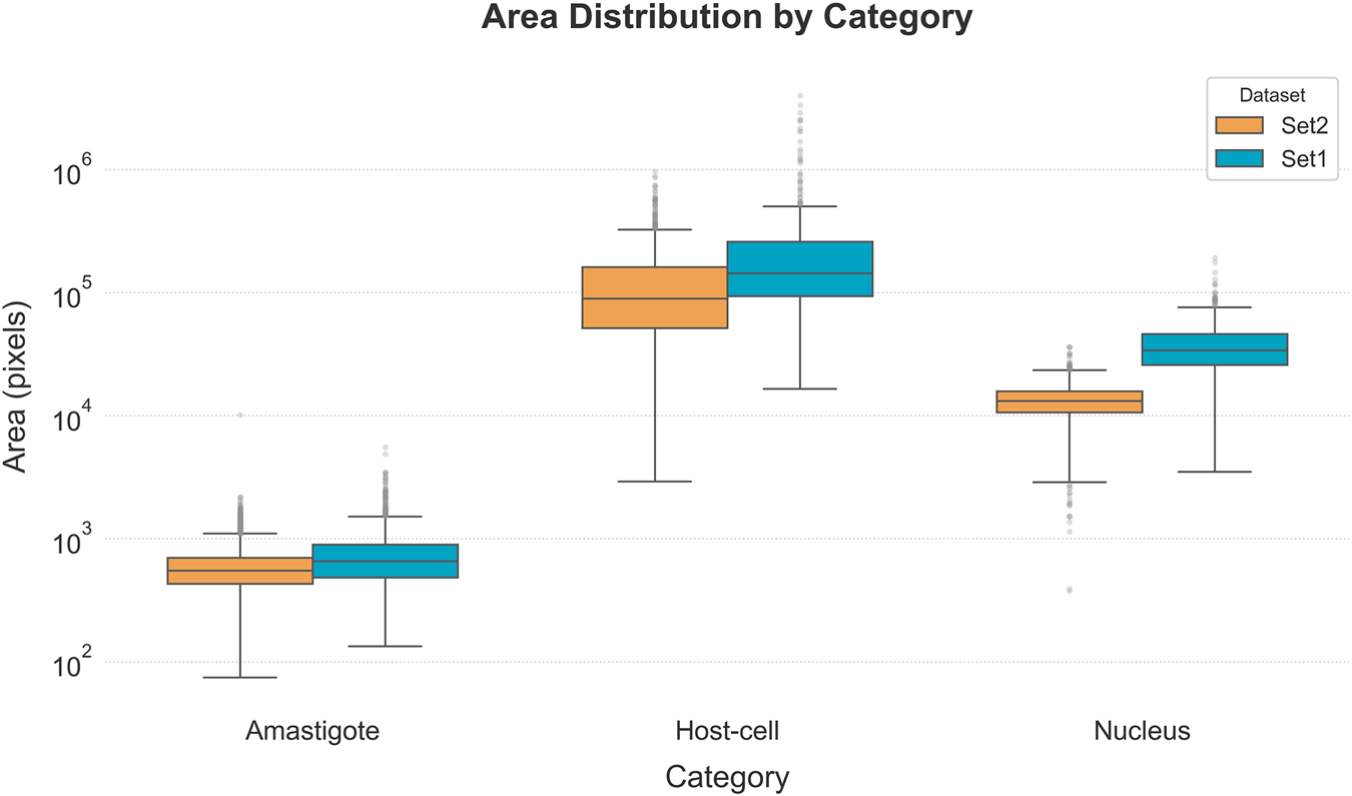
Area (pixels²) distribution of annotated objects by category in Set1 and Set2. Box plots comparing the pixel area distributions of amastigotes, host cells, and nuclei across both sets. Consistent size hierarchies are maintained (Host-cell > Nucleus > Amastigote) with Set2 showing marginally larger median sizes across all categories.

The object size analysis assessed through occupied areas in pixels highlighted distinctive patterns across both datasets. Notable differences were observed among the Host Cells class, where median areas in Set1 were 1.6-fold larger than those of Set2. Figures of 143.425 px² *versus* 89.239 px² with *p*-value < 0.001 were observed (Fig. 7). On the other hand, amastigotes demonstrated size consistency, with average areas of 776 ± 479 px² and 549 ± 315 px² for Set1 and Set2, respectively. Nuclei presented 5% outliers with areas > 2,500 px². These were primarily due to merged annotations in dense regions.

### Data splitting

Upon completion of the annotation process, we proceeded to data splitting for seamless use by AI models. We designed a strategy for a balanced splitting of the dataset towards an optimal training and evaluation of AI models. Given the small size of the two datasets Set1 and Set2 and the imbalanced distribution of amastigotes across different images, we implemented a data partitioning approach that shall maximize class balance and representativeness within each subset.

We employed a stratified random splitting based on amastigote count, ensuring that each subset maintained a proportional representation of amastigotes with varying image count. Following this strategy, we randomly divided the images of each set into three separate partitions training, validation, and test while preserving an approximate ratio of 70:20:10, resulting in respectively 64, 17 and 9 images in the training, validation, and test sets of each dataset.

### Model training and evaluation

To train the U-Net architecture for cellular objects segmentation, we implemented a patch-based processing pipeline to handle high-resolution images (1,844 × 2,709 pixels). Each image was divided into 24 non-overlapping grid patches (4 columns × 6 rows) using aspect ratio-preserving splitting with remainder distribution. Patches were resized and padded to 512 × 512 pixels using bilinear interpolation for images and nearest-neighbor for masks, with reflective padding for image boundaries. We applied a background filter to discard patches with less than 1% non-background pixels, significantly reducing class imbalance. Processed patches were normalized to [0,1] and masks were one-hot encoded into four classes: background, amastigotes (AM), host cells (HC), and nuclei (NU). We employed the Adam optimizer with an initial learning rate of 1e-4, implementing early stopping (patience = 12 epochs) and learning rate reduction on plateau (factor = 0.1, min_lr = 1e-6) to prevent overfitting. To address class imbalance, we adopted the Dice loss function during training. The model architecture incorporated L2 weight regularization (λ = 1e-4) and 50% dropout in decoder blocks for enhanced generalization. For evaluation, we computed standard multiclass segmentation metrics, including Dice coefficient, Intersection over Union (IoU), Precision, and Recall at the patch level, with results aggregated across all test set patches to provide comprehensive performance assessment. This patch-based evaluation strategy ensures rigorous quantification of segmentation quality while accommodating high-resolution image analysis.

In a second step, pretrained YOLOv8-n was trained on our microscopy images in the 1,024 × 1,024 pixels size to detect three classes, namely AM, HC, NU. We trained the model for 500 epochs, including 5-epoch linear warmup, with a batch size of 4. A systematic hyperparameter search was carried out. Automated tuning selected the optimizer AdamW after comparing several learning schedules and decay rates. Model’s performance was assessed through mAP50, Precision and Recall. All simulations, using both YOLOv8 and U-Net models, were conducted on a single NVIDIA Tesla T4 GPU on Google Colab.

### Performances: Object detection vs segmentation

**Fig. 8:**
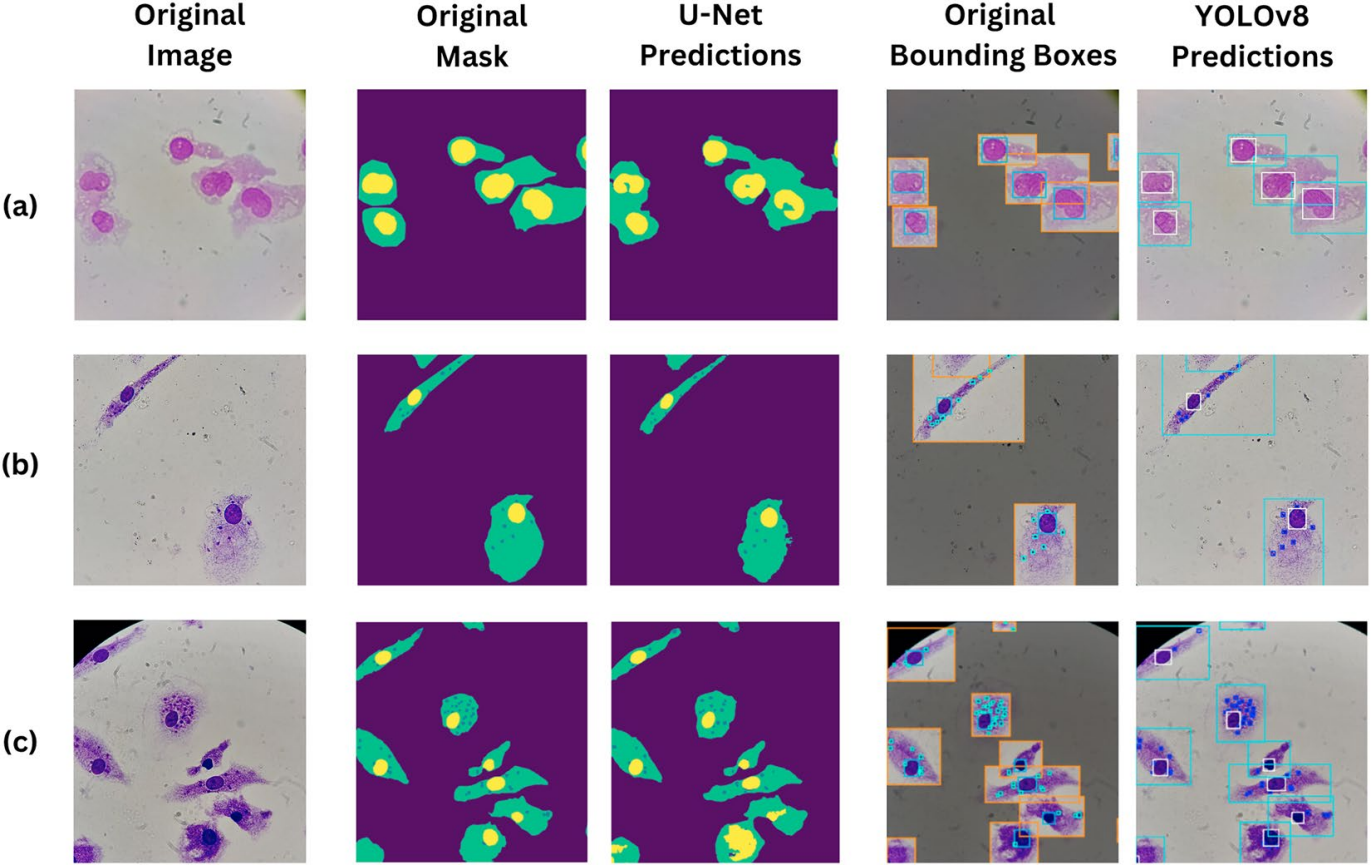
Comparative visualization of U-Net segmentation and YOLOv8 detection results on annotated microscopy images. From right to left, we provided the original image, the original polygonal mask followed by U-Net predicted masks, then the original bounding boxes and the YOLOv8 predictions. **(a)** An image example of uninfected cells demonstrating no false detection of macrophages, **(b)** An image example of low to moderate parasite load, and **(c)** An image example of high parasite load.

To compare the effectiveness of the segmentation and object detection approaches, we presented quantitative results from both models (Table 1) as well as a visual example of their outputs on microscopy images (Fig. 8). The U-Net model exhibited a Dice score of 75%, a Precision of 85% and a Recall of 71% on the test set, as for the amastigote class. These values compared closely to those obtained on the validation set, indicating stable performances of the model. Additionally, it achieved a IoU score of 62%, which reflects satisfactory concordance between original and predicted masks for each amastigote object. Furthermore, the YOLOv8 model also showed promising detection performance for the amastigote class (instances for “AM”), achieving a mAP50 of 83%, a precision of 86% and a recall of 71% on the test set. Similar results were observed on the validation set, with an mAP50 of 78%, a precision of83% and recall of 72%, indicating reliable detection across different image sets (Table 1), with 25.3 milliseconds per image latency.

**Table 1:** Comparative Performance of U-Net (Segmentation) and YOLOv8 (Detection) on Validation and Test Sets.

Images		U-NET				YOLOv8			
		IoU	Dice	Precision	Recall	Instances	mAP50	Precision	Recall
**Validation**									
ALL	34	0.77	0.79	0.87	0.82	2150	0.90	0.90	0.87
AM	34	0.77	0.64	0.83	0.74	1573	0.78	0.83	0.72
HC	34	0.76	0.86	0.84	0.89	269	0.95	0.91	0.93
NU	34	0.78	0.86	0.94	0.84	308	0.97	0.95	0.96
**Test**									
ALL	18	0.73	0.82	0.89	0.81	973	0.93	0.91	0.87
AM	18	0.62	0.75	0.85	0.71	690	0.83	0.86	0.71
HC	18	0.75	0.84	0.86	0.86	121	0.95	0.90	0.92
NU	18	0.82	0.88	0.96	0.85	126	0.99	0.96	0.98

Noticeably, performances on the HC, NU and ALL classes were significantly higher than those obtained for the AM class. This was expected, as per the small size of the amastigotes compared to the remaining objects within the images. Overall comparable performances could be obtained by both models, with the YOLOv8 being the most performing model in detecting and counting objects, while the U-Net provided significant value in delimiting objects and defining their shape, size and concomitance within the cellular context.

A visual comparison of representative microscopy samples processed by both models was shown in Fig. 8. From left to right, we presented the original microscopy image, the original mask created through manual annotation in Roboflow, the mask prediction of U-Net, the annotation masks converted into bounding boxes, and the object detection output from YOLOv8 appearing as labeled bounding boxes. This visual comparison illustrated how both models detected amastigotes while distinguishing them from host cells and nuclei. While U-Net provided detailed pixel-level segmentation masks of the different class instances (AM, HC, NU), YOLOv8 detected and localized objects using bounding boxes (Fig. 8). We observed consistent performances across images exhibiting low to moderate parasite densities (Fig. 8b), and high parasite densities (Fig. 8c). Noticeably, no false positive detections were observed on uninfected cells (Fig. 8a), supporting the reliability of the data annotation and the training process. To further assess the models performances in extreme low infection scenarios, we conducted an additional evaluation. Specifically, we simulated a case where we counted 112 cells (from 17 non annotated images), out of which only 1 cell was infected with 1 intracellular amastigote (infection rate ≈ 1:100). We then tested the trained and optimized YOLOv8 and U-Net models on these images. Both models successfully detected the single amastigote as a true positive, thus leading to no false negative detections (Supplementary Fig. S1, Table S1). It is noteworthy to point out that YOLOv8 generated 2 false positives, while U-Net only detected 1 false positive detection (Supplementary Table S1). These performances were obtained without data augmentation or extensive fine-tuning, suggesting that further specific optimization to low-burden detection could further enhance sensitivity and precision.

## Usage Notes

The AIR-LEISH^17^ dataset is designed for researchers working in parasitology, cellular biology, and computer vision and AI applications. It provides annotated microscopy images suitable for the training of deep learning models, such as YOLO, U-Net, R-CNN, among others. It supports a variety of use cases, including multiclass segmentation and object detection tasks through its adaptive annotations. Although we provided a case study that particularly focused on identifying intracellular parasites, the annotations included host cells and nuclei objects. Thus, the dataset can support cell-based image analysis tasks, such as quantifying cells, assessing infection levels or training AI tools for general cell segmentation and counting.

While the dataset is specifically designed for leishmaniases, its applicability could extend to other infectious diseases that involve macrophage-infecting pathogens with similar intracellular characteristics. Researchers working on other intracellular pathogens (e.g., *Toxoplasma gondii*, *Trypanosoma cruzi*, *Mycobacterium tuberculosis*) may also find the dataset and code useful for transfer learning applications. It can serve as additional data to train models to differentiate infected cells from non-infected cells. It can also serve as a supporting subset of an additional class of parasites to be detected by systems focusing on identifying and recognizing multiple types of parasites within mammalian cells.

## Supplementary information

Supplementary Information

## Data Availability

The datasets^17^ produced by this study are accessible on Zenodo under 10.5281/zenodo.17384855.

## Code availability

The code and detailed documentation to reproduce the results presented in this study are publicly available at https://github.com/Harigua/AI_leish_microscopy under the GNU General Public Licence v3.0.
