# Supplementary material for: AIR-LEISH: A Dataset of Giemsa-Stained Microscopy Images for AI-based Leishmania amastigotes Detection
Source: Sci Data. 2026 Feb 2;13:328. doi: 10.1038/s41597-026-06676-8 (PMC12966434; doi:10.1038/s41597-026-06676-8)
Supplement: Supplementary file 1 — Supplementary Information [file 41597_2026_6676_MOESM1_ESM.pdf]

Supplementary Information

Figure S1: Detection of low-parasite-burden cases using U-Net and YOLOv8 models

Representative example of an extremely low-infection scenario used to assess model performance. Among 112 manually screened macrophages, only one cell contained a single intracellular amastigote (see first panel). Both YOLOv8 and U-Net successfully detected the parasite (true positive), demonstrating sensitivity at extremely low parasite burden. From right to left, the panels show the original image, U-Net predictions, then the YOLOv8 predictions.

Original Image	U-Net Predictions	YOLOV8 Predictions
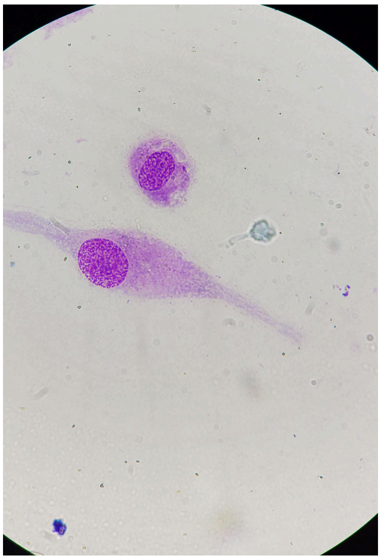	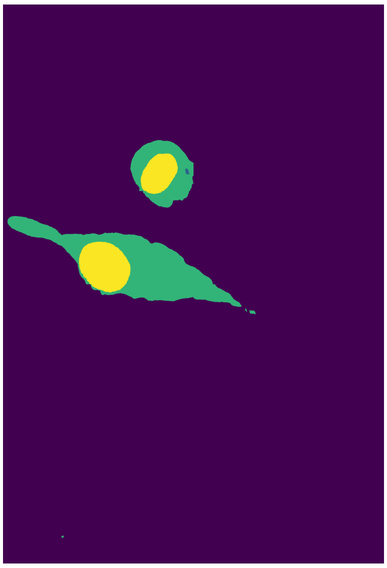	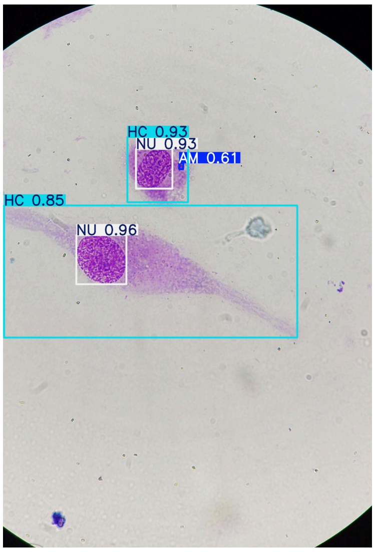
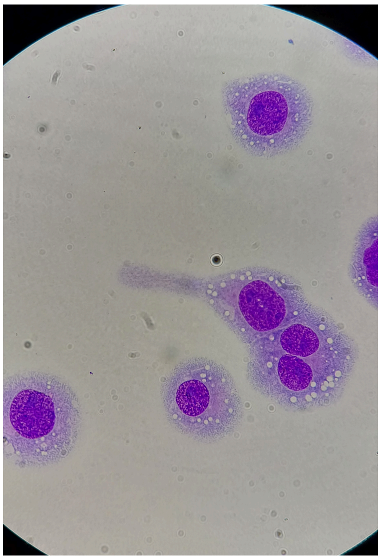	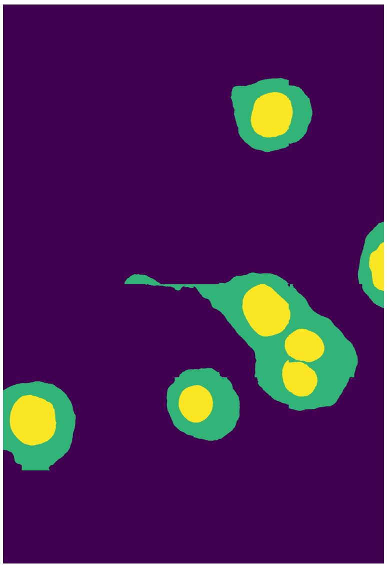	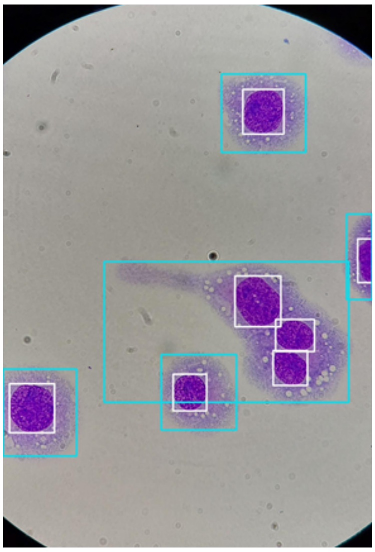

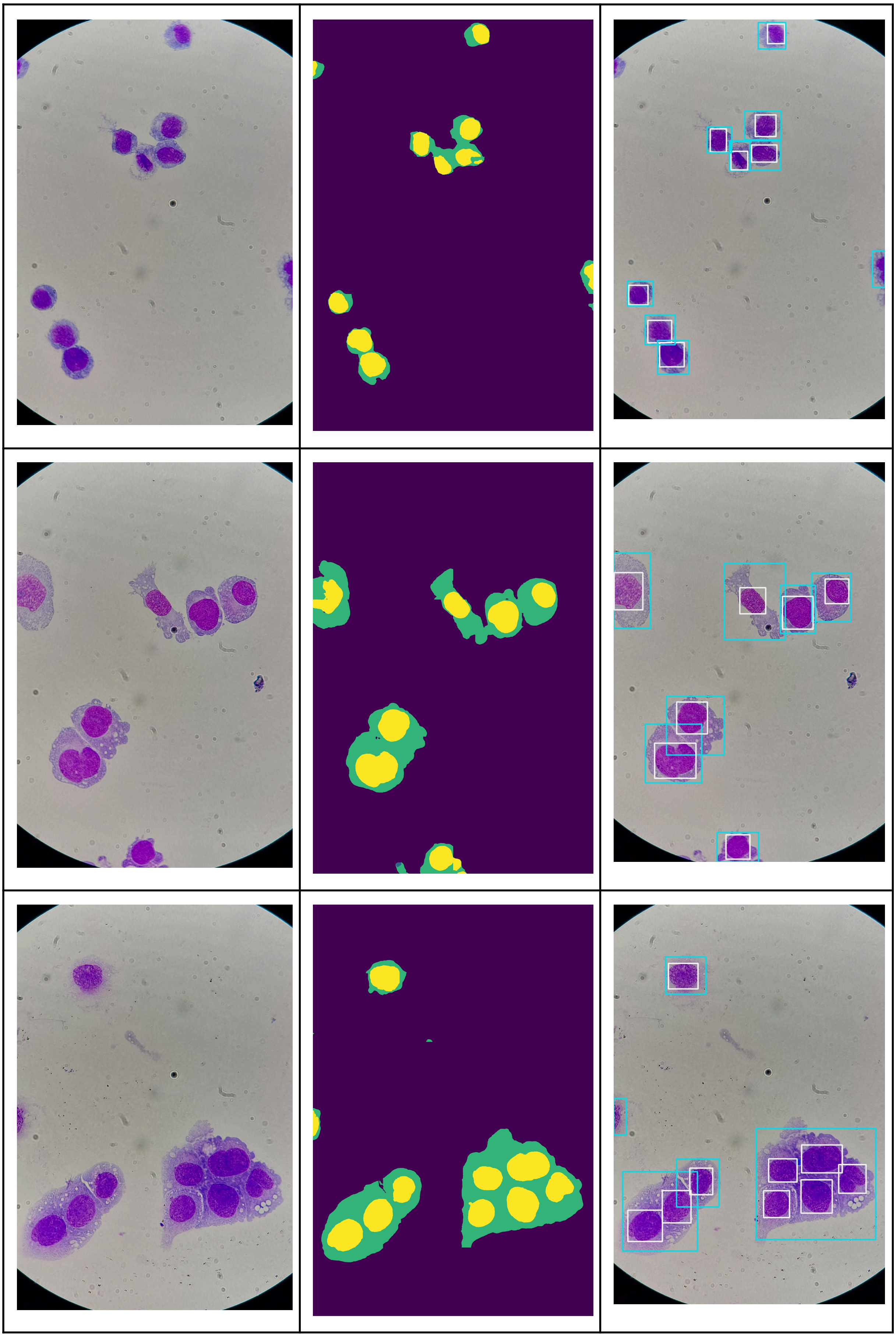

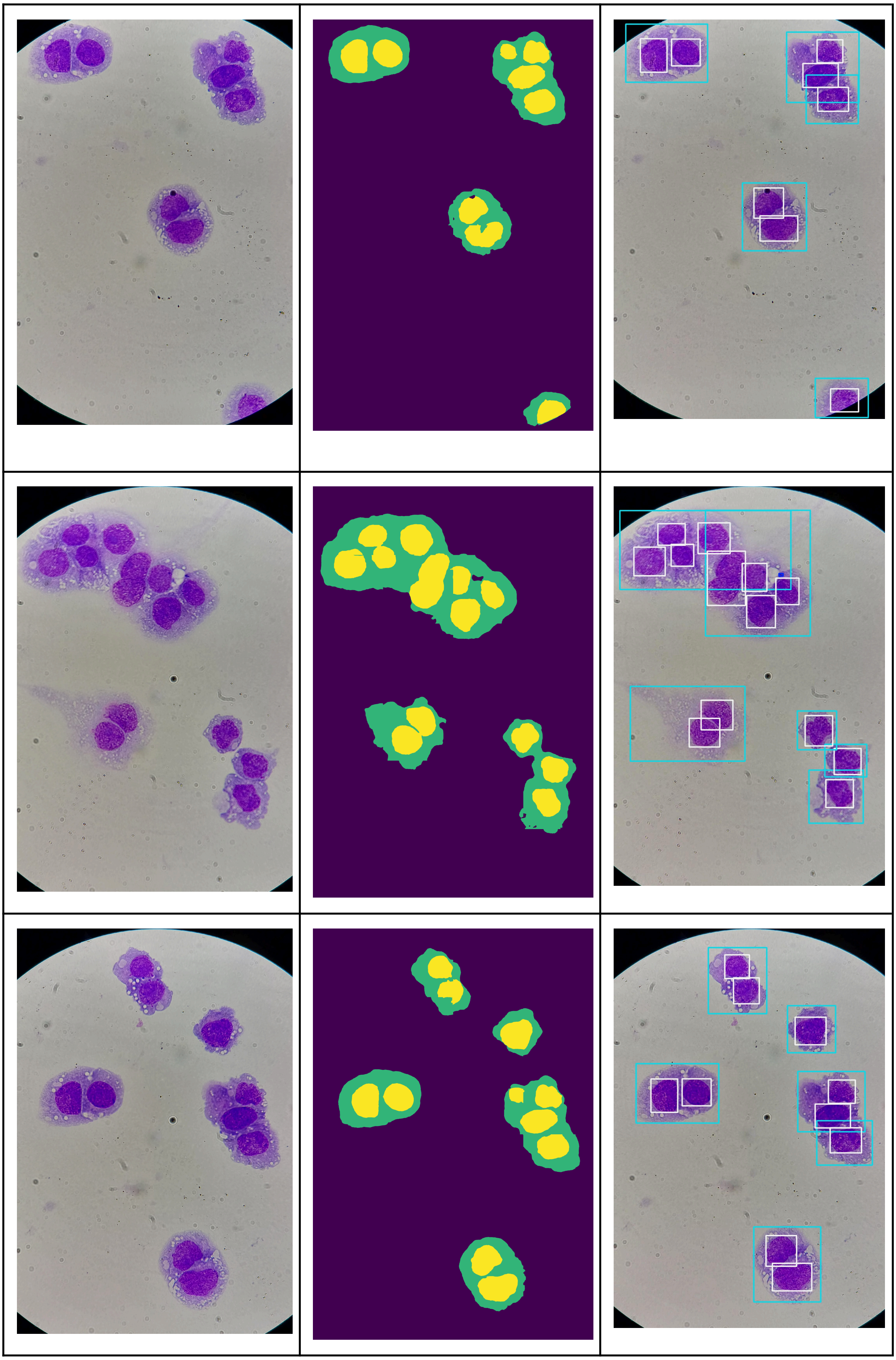

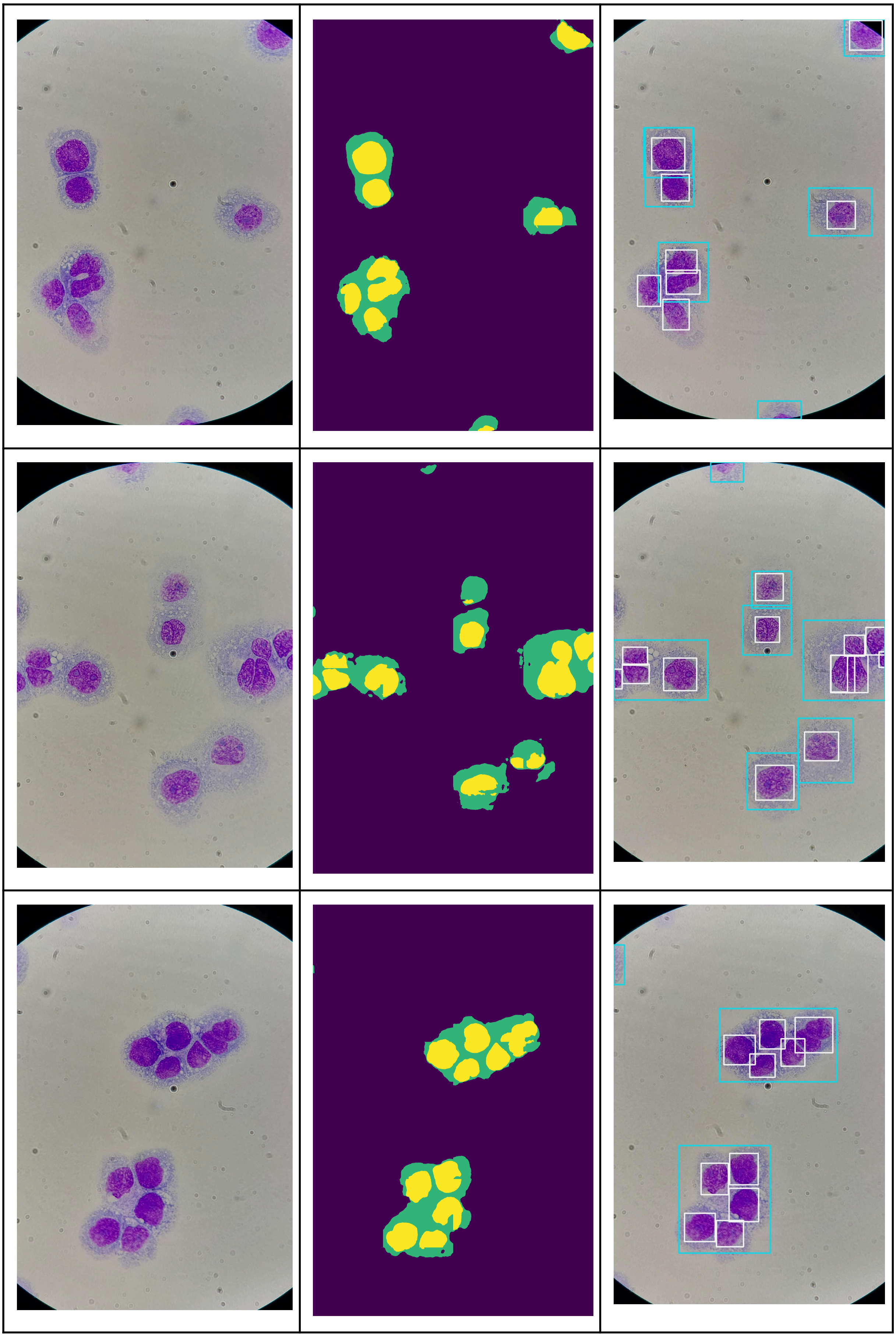

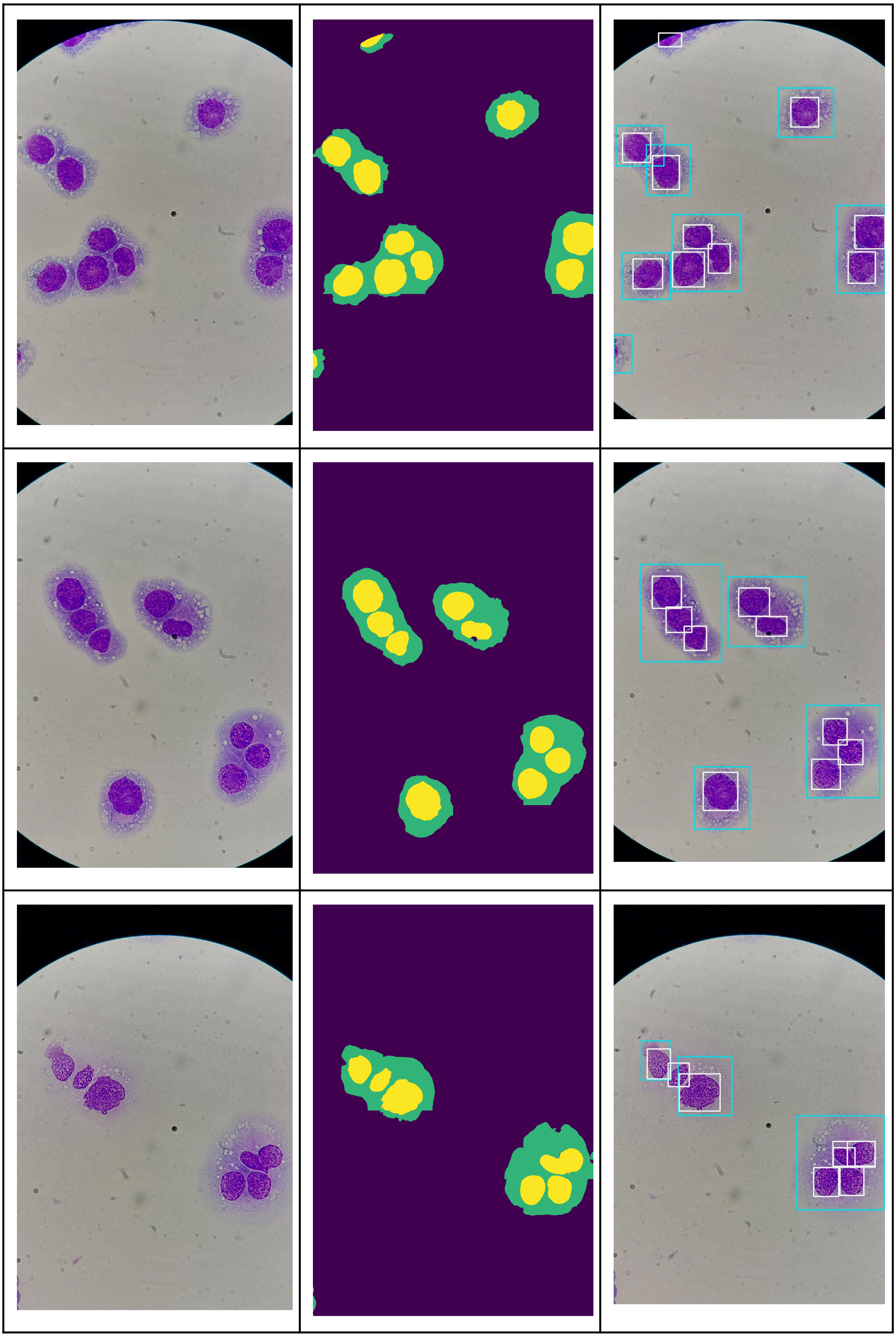

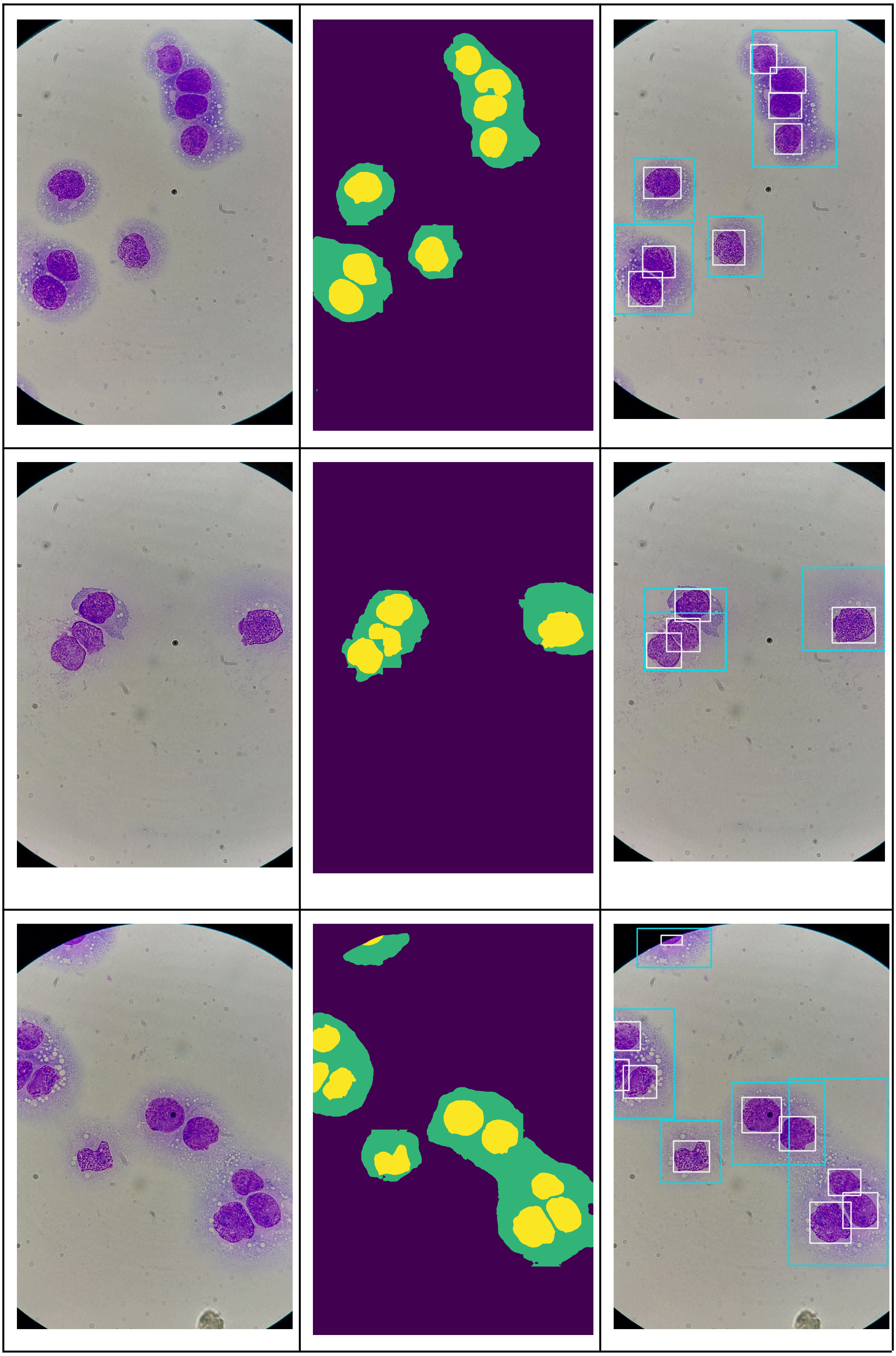

Table S1: Performance of YOLOv8 and U-Net under a simulated low infection scenario

	Expert annotation	U-Net *	YOLOv8
**Total Cells**	**112**	**-**	**87**
**Total Nucleus**	**142**	**-**	**145**
**True positives amastigotes (TP)**	**1**	**1**	**1**
**False negatives amastigotes (FN)**	**0**	**0**	**0**
**False positives amastigotes (FP)**	**0**	**1**	**2**

Summary of the detection performance of YOLOv8 and U-Net versus expert manual annotation when evaluated under a simulated low-infection rate (infection rate ≈ 1:100). A total of 112 macrophages were screened, among which only one cell was infected with a single intracellular amastigote. Both models successfully detected the parasite with no false-negative detections. U-Net generated 1 false-positive detections, while YOLOv8 generated 1 false-positive detections. These results confirm that both models maintain sensitivity in extremely low parasite burden conditions. (*) The U-net results do not provide cells, nucleus and amastigotes, as we used it for semantic segmentation.
